# Looking Back to Move Forward: The Current State of Research on the Clinical Applications of Camphor- and Menthol-Containing Agents

**DOI:** 10.7759/cureus.41426

**Published:** 2023-07-05

**Authors:** Don Hoang, Ashley Wong, Robert P Olympia

**Affiliations:** 1 Surgery, University of California Davis Medical Center, Sacramento, USA; 2 Internal Medicine, The George Washington University Hospital, Washington, D.C., USA; 3 Emergency Medicine and Pediatrics, Penn State Health Milton S. Hershey Medical Center, Hershey, USA

**Keywords:** upper respiratory tract infection, pruritus, pain management, complementary & alternative medicine, bibliometric analysis, menthol, camphor

## Abstract

Topically applied compounds containing camphor and menthol have been used to alleviate pain, cold symptoms, and pruritus, historically predominantly in East Asia. Being not studied well, they are less recognized in Western medicine. Given the commonality of pain, pruritus, and cold symptoms in addition to the growing need for non-opioid treatment options, the authors investigated clinical applications of such compounds for their over-counter usage. The purpose was to analyze current clinical research and applications regarding the use of these topical agents.

This study involved a bibliometric analysis of peer-reviewed articles, published in English and indexed in PubMed from 2010 to 2022, pertaining to camphor- and menthol-containing compounds. There were 103 results, of which 15 (14.6%) articles were related to the treatment of disorders related to health, such as upper respiratory infection, pain, and pruritus. Excluded were “non-research” articles (e.g., letters to the editor), articles that do not involve human subjects, reports of improper application or misuse (e.g., ingestion), and articles pertaining to intraoral, intranasal, and ophthalmic agents. Of these articles, the originating journals, respective journal impact factor scores, publication years, study designs, and study topics were identified. Underlying trends and themes regarding clinically relevant research on these compounds were subsequently discerned.

Based on this analysis, topical agents containing camphor and menthol are potentially effective at treating pain, upper respiratory infection symptoms, and pruritus in addition to potentially functioning as an antimicrobial. However, with a limited number of studies addressing these compounds' uses in each application, no definitive recommendation can be made regarding their use. Given the promising results of earlier studies, the authors recommend that more primary research, particularly randomized, double-blind controlled studies, be done regarding clinical applications of these substances.

## Introduction and background

Back pain, arthritis, upper respiratory infection (URI) symptoms, and pruritus are among the most common complaints in developed countries [1]. Currently, there are limited safe and effective treatments for pain, URI symptoms, and chronic pruritus [2-4]. Consequently, research on alternative therapies and management strategies for these issues needs to be conducted.

Topical agents have emerged as promising alternative or complementary modalities of treatment for pain, pruritus, and URI symptoms over time [3,5,6]. Often lacking significant systemic effects, topical medications tend to be safer, have less addictive potential, and are more accessible than oral and parenteral therapies [7]. As such, topical medications are an appealing treatment option and have become a topic of research regarding the management of pain, pruritus, and URI symptoms.

The authors considered existing remedies that may not be as well recognized as the other topical agents. Camphor- and menthol-containing agents, in particular, stood out. From a pain standpoint, camphor activates TRPV1 and inhibits TRPA1, on which menthol also has a bimodal action, resulting in an analgesic effect [8]. For URI symptoms, camphor and menthol both activate inhibiting transient receptor potential (TRP) channel M8, which is believed to inhibit respiratory reflexes to alleviate irritation and cough. These compounds have deep roots in Eastern medicine and have historically been used for alleviating pain, cold symptoms, and pruritus in various cultures. However, as these compounds are not as well recognized in Western medicine, clinical research is sparse.

The bibliometric study seeks to evaluate the current research that exists on the clinical applicability of compounds containing camphor and menthol and make recommendations for future research regarding their use. This involved a comprehensive review of articles found in PubMed since 2010. McGranahan & Parker noted efforts to address the opioid epidemic starting in the early 2010s with opioid drug reformulation and declining prescription rates; as such, 2010 was chosen as the start date of the analysis [9]. The authors aimed to present general conclusions about these substances' application in clinical medicine and provide direction for future research regarding these topical compounds. This article was previously presented as a poster at PAINWeek 2022 on September 8, 2022.

## Review

Methods

This study involves a bibliometric analysis of English, peer-reviewed articles indexed in PubMed from 2010 to 2022 using the search term "Menthol AND Camphor”. The journal, the journal impact factor score (acquired from the 2021 Journal Citation Reports), "clinical relevance," year of publication, study design, and study topic of each of the resulting articles were identified. Underlying themes regarding research on camphor- and menthol-containing compounds were reviewed independently by DH and AW, discrepancies were reviewed, and overarching themes were decided upon following discussion. Trends in research were evaluated by comparing the number of relevant publications from year to year and displayed using a bar graph. For the purpose of this analysis, “clinically relevant” articles included those that related to the treatment of disorders related to health. Articles that were not primary or secondary research (e.g., letters to the editor), articles that do not involve human subjects, reports of improper application or misuse (e.g., ingestion), and articles pertaining to intraoral, intranasal, and ophthalmic agents were excluded. Ultimately, counts of journals, study designs, and study topics were gathered, and the distribution of publication years was analyzed and displayed using a histogram.

Bibliometric analysis

The PubMed search generated 103 results, of which 15 (14.6%) articles were deemed “clinically relevant” research by the authors using the criteria discussed above. Table 1 lists all the clinically relevant articles and the specifics of the bibliometric analysis. Each article was published in a unique journal. With regard to the Journal Impact Factor, the range was 0.863 to 16.876, with an average of 4.212 and a median of 3.335.

**Table 1:** Bibliometric analysis of “clinically relevant” articles URI: Upper respiratory infection

Article	Author(s), Year	Journal	Journal Impact Factor (According to the 2021 Journal Citation Reports)	Study Design	Topic
A prospective study examining the effect of selected topical and systemic drugs on Pruritus Grading System Score and STAT 6 Expression in patients of prurigo nodularis	Agrawal et al., 2021 [10]	Indian Journal of Dermatology	1.757	Prospective Case Series	Treatment of pruritus
Treatment of the common cold	DeGeorge et al., 2019 [3]	American Family Physician	5.305	Review	Treatment of URI symptoms
Topical therapies for knee osteoarthritis	Rodriguez- Merchan, 2018 [11]	Postgraduate Medicine	4.379	Review	Treatment of pain
Use of camphor and essential oil balms for infants in Cambodia	Bazzano et al., 2017 [12]	Journal of Tropical Pediatrics	1.794	Qualitative Case Study	Adverse effects
Clinical efficacy of polyherbal formulation Eezpain spray for muscular pain relief	Nawaz et al., 2015 [13]	Pakistan Journal of Pharmaceutical Sciences	0.863	Prospective Case Series	Treatment of pain; anti- inflammatory activity
Evaluation of treatments for pruritus in epidermolysis bullosa	Danial et al., 2015 [14]	Pediatric Dermatology	1.997	Cross-Sectional Study	Treatment of pruritus
Camphor induces cold and warm sensations with increases in skin and muscle blood flow in human	Kotaka et al., 2014 [15]	Biological and Pharmaceutical Bulletin	2.264	Non-Randomized Controlled Study	Improving circulation
Efficacy and safety of topical Trikatu preparation in, relieving mosquito bite reactions: a randomized controlled trial	Maenthaisong et al., 2014 [16]	Complementary Therapies in Medicine	3.335	Double-Blinded Randomized Controlled Trial	Treatment of pruritus; Anti-inflammatory activity
Prevention and treatment of the common cold: making sense of the evidence	Allan & Arroll, 2014 [17]	Canadian Medical Association Journal	16.876	Review	Treatment of URI symptoms
A comparative evaluation of local application of the combination of eutectic mixture of local anesthetics and capsaicin for attenuation of venipuncture pain	Gupta et al., 2013 [18]	Anesthesia & Analgesia	6.627	Double- Blinded Randomized Controlled Trial	Treatment of pain
Menthol differs from other terpenic essential oil constituents	Kolassa, 2013 [19]	Regulatory Toxicology and Pharmacology	3.598	Review	Adverse effects
Therapeutic options for acute cough due to upper respiratory infections in children	Paul, 2012 [20]	Lung	3.777	Review	Treatment of URI symptoms
Tea tree oil attenuates experimental contact dermatitis	Wallengren, 2011 [21]	Archives of Dermatological Research	3.033	Non- Randomized Controlled Trial	Treatment of pruritus; Anti-inflammatory activity
Novel treatment of onychomycosis using over-the-counter mentholated ointment: a clinical case series	Derby et al., 2011 [22]	Journal of the American Board of Family Medicine	2.395	Prospective Case Series	Antimicrobial activity
Contact dermatitis to Vicks VapoRub	Noiles & Pratt, 2010 [23]	Dermatitis	5.185	Case Report	Adverse effects

**Figure 1:**
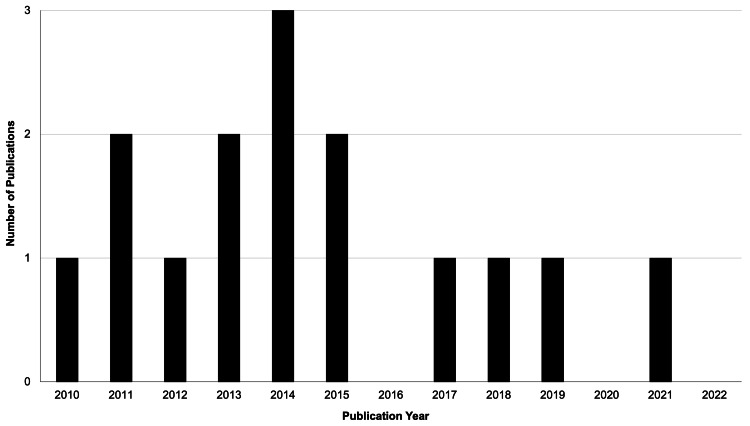
Number of "clinically relevant" publications per year since 2010

The publication counts per year demonstrated no obvious trends in the total number of publications over time, with a maximum of three articles in 2014, or the type of journal to which these articles are published. Figure 1 displays the trend in publication counts over time.

Of the total 15 studies included in this review, five were reviews (33.3%), three were prospective case series (20.0%), two were non-randomized controlled studies (13.3%), two were randomized, double-blind controlled studies (13.3%), one was a cross-sectional study (6.7%), one was a qualitative case study (6.7%), and one was a case report (6.7%). Regarding discussed topics, pruritus in four articles (26.7%), adverse effects were discussed in three articles (20.0%), pain in three articles (20.0%), inflammation in three articles (20.0%), cold symptoms in three articles (20.0%), circulation effects in one article (6.7%), and antimicrobial activity in one article (6.7%). Table 2 summarizes the specific conclusions for each of these articles.

**Table 2:** Summary of the conclusions of each “clinically relevant” article NSAIDs: Non-steroidal anti-inflammatory drugs

Article	Author(s), Year	Conclusion
A prospective study examining the effect of selected topical and systemic drugs on Pruritus Grading System Score and STAT 6 Expression in patients of prurigo nodularis	Agrawal et al., 2021 [10]	Combinations of oral (e.g., antihistamines, nortriptyline, thalidomide) and topical agents (e.g., camphor + menthol, steroids, emollients, salicylic acid) can help control prurigo nodularis.
Treatment of the common cold	DeGeorge et al., 2019 [3]	Effective treatments for cold symptoms in adults include over-the-counter (OTC) analgesics, zinc, nasal decongestant, and ipratropium. Lactobacillus casei has lower-quality evidence in older adults. In children, acetylcysteine, honey, nasal saline irrigation, intranasal ipratropium, and topical ointment containing camphor, menthol, and eucalyptus oils are considered safe and effective.
Topical therapies for knee osteoarthritis	Rodriguez- Merchan, 2018 [11]	Current management guidelines recommend topical NSAIDs for knee osteoarthritis (OA) treatment, as they have fewer gastrointestinal complications compared with oral NSAIDs. The role of other topical therapies (e.g., menthol and creams containing glucosamine sulfate, chondroitin sulfate, and camphor) needs further research but has shown a low level of evidence for pain mitigation.
Use of camphor and essential oil balms for infants in Cambodia	Bazzano et al., 2017 [12]	Topical products, containing camphor, menthol, and eucalyptus, were commonly used in infants in Cambodia. Parents and caregivers should be educated on the risks of camphor- and menthol-containing products.
Clinical efficacy of polyherbal formulation Eezpain spray for muscular pain relief	Nawaz et al., 2015 [13]	Eezpain spray (consisting of gaultheria oil, eucalyptus oil, turpentine oil, clove oil, menthol, and camphor) has shown efficacy in mild to moderate cases on applying on affected parts.
Evaluation of treatments for pruritus in epidermolysis bullosa	Danial et al., 2015 [14]	The most effective treatments in treating pruritus in epidermolysis bullosa included creams, topical prescription corticosteroids, oils, oral hydroxyzine, topical diphenhydramine, and vaporizing rub (menthol, camphor, eucalyptus). Randomized controlled trials are recommended for additional evaluation.
Camphor induces cold and warm sensations with increases in skin and muscle blood flow in human	Kotaka et al., 2014 [15]	Camphor and menthol each resulted in increases in local blood flow in skin and muscle. Camphor induces both cold and warm sensations while menthol just induces a cold sensation.
Efficacy and safety of topical Trikatu preparation in, relieving mosquito bite reactions: a randomized controlled trial	Maenthaisong et al., 2014 [16]	The combination of Trikatu extract, camphor, menthol, and eucalyptus does not have additional anti-inflammatory effects compared to a control of camphor, menthol, and eucalyptus, which has been shown to relieve insect bite reactions.
Prevention and treatment of the common cold: making sense of the evidence	Allan & Arroll, 2014 [17]	Antihistamine monotherapy has no clinically meaningful benefit, but combination therapy is beneficial in adults and older children. Decongestant has small benefit but uncertain clinical significance. Intranasal ipratropium has probable benefits. OTC cough treatment (e.g., antitussives, expectorants) has unclear benefit in adults and no benefit in children. Vapor rubs (containing camphor, menthol, and eucalyptus oil) have unclear benefits and demonstrated harm based on a “poorly described randomized, blinding limited, single study”. NSAIDs are likely beneficial for pain. Acetaminophen is likely effective for fever and analgesia. Antibiotics have no benefits and clear harms.
A comparative evaluation of local application of the combination of eutectic mixture of local anesthetics and capsaicin for attenuation of venipuncture pain	Gupta et al., 2013 [18]	Combination of capsaicin (e.g., Myolaxin ointment containing oleoresin capsaicin, methylsalicylate, menthol, camphor, and eucalyptus oil) and eutectic mixture of local anesthetics is not significantly more effective than individual drugs alone, which demonstrate significant improvement in pain.
Menthol differs from other terpenic essential oil constituents	Kolassa, 2013 [19]	While convulsions have been seen and can be explained in camphor, thujone, and eucalyptus oil overdose/poisoning, no convulsions have been seen or explained in menthol overdose/poisoning. Therefore, menthol’s side effect profile is not comparable to those of other terpenic derivatives.
Therapeutic options for acute cough due to upper respiratory infections in children	Paul, 2012 [20]	While there is not strong evidence supporting the use of narcotics OTC oral antitussives, and expectorants for upper-respiratory associated pediatric cough, honey, and topically applied vapor rubs (camphor, menthol, and eucalyptus oils in a petrolatum base) may be effective.
Tea tree oil attenuates experimental contact dermatitis	Wallengren, 2011 [21]	While compounds containing zinc, menthol and camphor have historically been used to relieve itching, no difference was found in the perception of itch from histamine.
Novel treatment of onychomycosis using over-the-counter mentholated ointment: a clinical case series	Derby et al., 2011 [22]	Vicks VapoRub (containing thymol, menthol, camphor, and oil of eucalyptus) showed positive clinical effects in treating onychomycosis.
Contact dermatitis to Vicks VapoRub	Noiles & Pratt, 2010 [23]	Vick's VapoRub (containing turpentine oil, eucalyptus oil, cedar leaf oil, camphor, menthol, nutmeg oil, and thymol) was demonstrated to directly cause acute contact dermatitis in a 66-year-old female.

Discussion

Camphor- and menthol-containing substances have been documented for their topical use for acute and chronic pain, pruritus, and cold symptoms relief as well as antimicrobial purposes. Proposed analgesic implementation included knee osteoarthritis [11], myalgias, neuralgias [13], and venipuncture pain [18], through possible improvements in blood circulation [15] among other mechanisms. These agents have shown potential in improving pruritus [10,14] through potential anti-inflammatory effects [16], but contradictory evidence does exist [21].

Other benefits of these agents include relieving nasal congestion and reducing nighttime cough frequency and severity, improving sleep for both the child and parents [3,17,20]. Additionally, antimicrobial properties were observed against onychomycosis [22]. Several of the excluded, “non-clinically relevant” articles (i.e., not involving human subjects) discussed other possible antimicrobial effects of menthol and camphor; however, they were not included in this analysis as they failed to meet criteria.

Potential side effects of these topical agents include contact dermatitis [23], allergic reactions, and camphor/menthol poisoning due to accidental ingestion, which could cause symptoms such as seizures [12,19]. That said, camphor, not menthol, is responsible for convulsions, so each compound's side effect profile should be considered separately [19]. 

## Conclusions

Topical compounds containing camphor and menthol have traditionally been used to alleviate pain, cold symptoms, and pruritus, especially in East Asian cultures. As these symptoms are fairly common, this bibliometric analysis aimed to assess the presence of peer-reviewed publications to better guide evidence-based, clinical applications of these agents. As PubMed was the only search engine utilized, relevant literature from other platforms may have been excluded from the analysis. Similarly, while the authors tried to include all research pertaining to topical camphor- and menthol-containing agents with the non-specific search terms, research articles referencing brand names of different formulations of these compounds, such as Tiger Balm, Eagle Balm, Vicks VapoRub, and Bengay, may have been excluded.

Based on existing literature, compounds containing camphor and menthol demonstrate potential in relieving pain, alleviating cold symptoms, lessening pruritus, and even functioning as an antimicrobial. Given the limited number of studies, however, a definitive recommendation cannot be made regarding their use. The authors recommend additional randomized, double-blind controlled studies be done to address the clinical application of these substances, particularly in the contexts of relieving localized pain, localized pruritus, and URI symptoms.
